# Is It Worth While to Try to Save the Teeth?

**Published:** 1902-03-15

**Authors:** Samuel A. Hopkins


### IS IT WORTH WHILE TO TRY TO SAVE THE TEETH?

BY SAMUEL A. HOPKINS, M.D., D.D.S.

Extract from a paper entitled "Science as a Teacher of Prophylaxis," published in the February *International Journal*.

We are doing our work in a comfortable way, showing more or less skill and doing more or less good, and obtaining a comfortable income. Why excite ourselves about the rest of the world? If we are here to be content, eat, drink and be merry; it may not be worth while, but, thanks be to God, that is not the motive that actuates the professional man in dentistry. Ours is one of the professions in which men are continually striving to cast off the sources of their incomes by substituting prevention for cure. Scamps and charlatans belong to all professions, but the proportion in dentistry is relatively small. Our work is hard and exacting; the dishonest and loose-fibered man can do better for himself in some other calling. Therefore, I feel that it is only necessary to show that this work of preventing caries is feasible, that it is important to the improvement of the human race, and that a fair degree of success awaits our efforts, to have every honorable member of the profession do his part to bring about so desirable a reformation.

When I make the assertion that the development of the dental organs and the strength of the same is and always will be in ratio to their use; and when I assert that lack of use will infallibly tend to weaken, if not to the suppression of these organs, I am simply stating a well-known scientific principle. I do not pretend to know what the intention of the Almighty is in regard to the future of the human race, nor do I believe any one else does ; but if we conscientiously believe that in the process of evolution the teeth are doomed, then it is certainly foolish to waste our time and energies in combatting degeneracy. It may rather be wiser to help on the process by wholesale extraction, that we may at least have clean mouths and gums, and thereby lessen the dangers of general infection from disease of the teeth. This thought may be repulsive, but it is not illogical.

As a matter of fact, strong teeth are associated with and are essential to the best types of manhood. As a result of the examination of school children in this and other countries, it has been shown that there does exist a correspondence in physical soundness and mental acuteness in children which corresponds relatively to the integrity of the teeth. It is true that this ratio is not pronounced nor constant, but I firmly believe that it will be more emphatically demonstrated when these investigations shall have been carried further.

The child with strong teeth will be found to have decidedly the best of it, both physically and mentally, and that he will be morally stronger will follow as a matter of course. As the child advances in age, the differences become marked, and as we reach the age when boys enter college this fact becomes established in a remarkable degree. It has been my fortune to have for patients a large number of college students, and to be brought into professional and social contact with trainers, coaches and captains of crews and teams, and have had exceptional opportunities for examining the teeth of college athletes. I have not been able to tabulate the results of such experience nor to classify it, as oftentimes the examinations have been hurriedly made, but from a general summary the conditions are most striking. The proportion of good, strong sets of teeth in such men is greatly above the average, as may be observed by the most superficial examination.

It can not be denied that oftentimes great intellectuality is associated with inferior dental conditions, and men of large physique frequently have poor teeth; but that combination of vital energy and mental strength which goes to make commanders and vigorous pioneers is rarely found associated with frail teeth. If, then, deterioration of the dental organs is a potent influence in the deterioration of the race, are we not morally bound to exert ourselves to combat so great a menace in every possible way?

